# Intraoperative tracking of tissue perfusion during cerebral aneurysm surgery with laser speckle contrast imaging: insights beyond standard intraoperative neuromonitoring for detecting ischemia

**DOI:** 10.1117/1.NPh.13.3.035003

**Published:** 2026-07-07

**Authors:** Ebenezer Raj Selvaraj Mercyshalinie, Abdurrahman F. Kharbat, Michael Machiorlatti, Andrew K. Dunn, Andrew Bauer, Christopher S. Graffeo, David R. Miller

**Affiliations:** a The University of Oklahoma, Stephenson School of Biomedical Engineering, Norman, Oklahoma, United States; b University of Oklahoma College of Medicine, Department of Neurosurgery, Oklahoma City, Oklahoma, United States; c University of Oklahoma Health Sciences Center, Department of Biostatistics and Epidemiology, Oklahoma City, Oklahoma, United States; d The University of Texas at Austin, Department of Biomedical Engineering, Austin, Texas, United States

**Keywords:** laser speckle contrast imaging, cerebral blood flow, ischemia, stroke, neurosurgery

## Abstract

**Significance:**

Intraoperative cerebral ischemia is a critical complication that can arise during cerebral aneurysm clipping surgery. Although intraoperative neuromonitoring (IONM) can detect resulting functional deficits, these alerts may occur minutes after the initial ischemic event.

**Aim:**

We aimed to demonstrate the clinical utility of laser speckle contrast imaging (LSCI) for continuous, real-time monitoring of cerebral blood flow (CBF) during aneurysm surgery.

**Approach:**

In this case study, a 67-year-old female underwent a craniotomy for a left-sided cerebral aneurysm. A microscope-integrated LSCI system was used to continuously monitor cortical perfusion. After surgery, LSCI data were correlated with IONM alerts, indocyanine green angiography (ICGA), and surgical events, including an ischemic period following clip placement. A paired t-test, mixed effects model, and changepoint analysis were used to compare mean perfusion between the pre-ischemic and ischemic periods across seven regions of interest (ROIs) on the cortical surface.

**Results:**

LSCI detected a widespread drop in cortical perfusion immediately following the application of an aneurysm clip. This perfusion deficit was detected by LSCI ∼8  min before the corresponding IONM alert. During the ischemic period, blood flow decreased across six of the seven ROIs, with reductions ranging from 7.9% to 28.0%. The overall decrease in perfusion from the pre-ischemic to the ischemic period was statistically significant (p<0.0001).

**Conclusions:**

LSCI can provide continuous imaging of tissue perfusion during surgery, enabling the detection of developing ischemia before functional deficits may be evident on IONM. The ability of LSCI to track tissue perfusion changes highlights its potential as a valuable complementary tool for enhancing surgical safety alongside IONM and ICGA.

## 1 Introduction

The primary objective of surgical intervention for intracranial aneurysms is the complete obliteration of the aneurysm from the circulation while preserving the patency of all adjacent parent, branching, and perforating arteries.^1^^,^^2^ A major source of morbidity and mortality associated with these procedures is iatrogenic cerebral ischemia, which can result from the inadvertent compromise of blood flow during clip application or mobilization of cerebral vascular structures.^3^^,^^4^ Even planned, temporary clipping of vessels to facilitate dissection carries a time-dependent risk of causing neurological deficits, underscoring the critical need for precise and continuous monitoring of cerebral blood flow (CBF) throughout the operation.^5^ Maintaining adequate tissue perfusion is critical to prevent postoperative complications and to ensure favorable patient outcomes.^6^

Intraoperative neuromonitoring (IONM), which encompasses a range of modalities including somatosensory evoked potentials (SSEPs) and motor-evoked potentials (MEPs), plays a crucial role in intraoperative functional assessment of neural pathways and detection of evolving neurological injury.^7^ IONM is valuable for detecting potential neurological damage such as an ischemic insult; however, signal changes often occur minutes after an ischemic insult has become severe enough to cause neuronal dysfunction.^8^^,^^9^ Indocyanine green angiography (ICGA), while offering high-quality visualization of vessel patency,^10^^,^^11^ provides only qualitative, intermittent snapshots of CBF rather than continuous quantitative CBF visualizations, and its view can be obscured by anatomical structures or incomplete dye filling. Visual assessment through a surgical microscope and ICGA are the primary intraoperative clinical standard for evaluating tissue viability and perfusion;^12^ however, subtle changes in tissue perfusion are not readily apparent through visual inspection (via the microscope optics or via conventional camera-based imaging) of the cortical surface alone, and interpretations of perfusion with ICGA are qualitative and subjective.^13^ Further, attempts for more quantitative perfusion methods with ICG video angiography are not yet standardized nor widely clinically adopted.^12^ These limitations create a critical gap in the utility of IONM and ICGA and call for a monitoring technology that can offer continuous, quantitative, and direct visualization of cortical perfusion throughout the entire surgical procedure.

Laser speckle contrast imaging (LSCI) has emerged as a promising optical technology to address this unmet clinical need for continuous CBF monitoring.^14^^–^^17^ LSCI is a noninvasive imaging technique that provides real-time, high-resolution visualization of microvascular blood flow with high spatiotemporal resolution.^18^ The technique works by illuminating the tissue with coherent laser light and analyzing the resulting speckle pattern; the motion of red blood cells within the blood vessels causes this pattern to blur, and the degree of blurring is processed to generate a semi-quantitative map of blood flow.^19^^,^^20^ LSCI can be integrated on a surgical microscope to enable uninterrupted data acquisition throughout the surgical procedure,^21^^,^^22^ enabling real-time and continuous detection of blood flow changes as they occur.^23^^,^^24^ LSCI can provide semi-quantitative units of blood flow and objectively assess changes in blood flow relative to baseline values.^25^^–^^29^ The noncontact, label-free nature of LSCI makes it ideally suited for integration into the neurosurgical workflow. The imaging system can be positioned to provide continuous monitoring without interfering with surgical access or requiring interruption of the procedure for assessment.^23^^,^^24^^,^^30^ This represents an advantage over other monitoring modalities that may require periodic repositioning or activation. By not requiring a dye injection, LSCI allows for uninterrupted perfusion assessment during critical surgical maneuvers such as temporary clipping, dissection around perforating arteries, or the application of permanent clips.^21^^,^^31^^–^^33^

The aim of this study is to present a detailed case report demonstrating the clinical utility of LSCI continuous monitoring during craniotomy for aneurysm clipping. Specifically, we sought to determine if continuous LSCI monitoring could detect clinically significant perfusion changes in real-time throughout the procedure. To our knowledge, this represents the first report of a microscope-integrated LSCI system being used to monitor and quantify an iatrogenic ischemic event during neurosurgery. This case provides direct evidence supporting the feasibility of continuous LSCI monitoring for detecting evolving ischemia during neurovascular procedures.

## 2 Materials and Methods

**Fig. 1:**
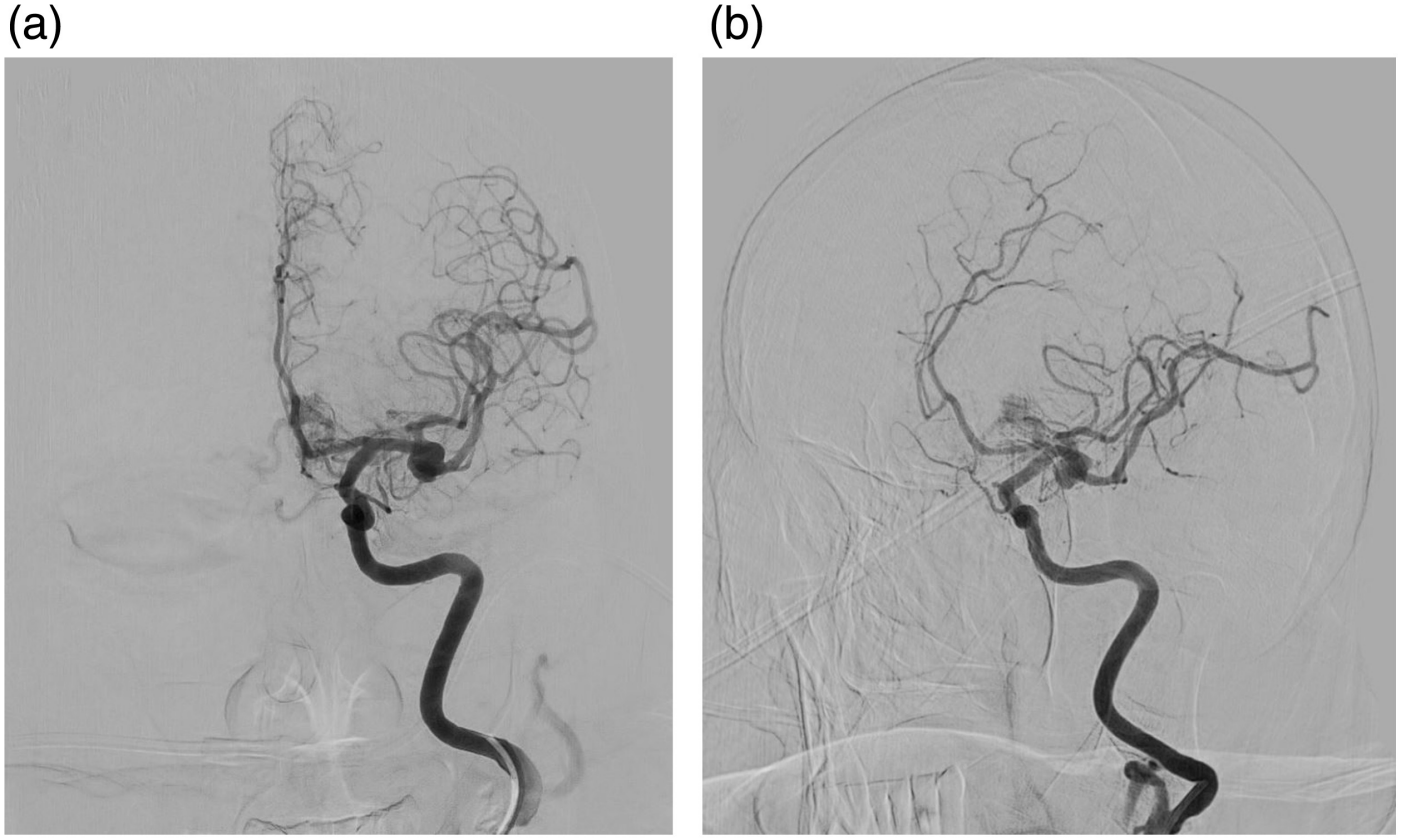
(a) Anteroposterior and (b) oblique views of diagnostic cerebral angiograms utilizing biplane fluoroscopy and contrast injections of a 67-year-old female with a history of a prior aneurysm clipping. The patient presented with a newly discovered, asymptomatic left-sided middle cerebral artery (MCA) aneurysm.

This study details the application of microscope-integrated LSCI for continuous real-time monitoring of cortical perfusion during cerebral aneurysm clipping surgery. The clinical study was approved by the Institutional Review Board of the University of Oklahoma Health Sciences Center (IRB #18049) and performed in accordance with the Strengthening the Reporting of Observational Studies in Epidemiology (STROBE) guidelines.^34^ Written and informed consent was obtained from the patients prior to surgery. We performed clinical measurements during seven cerebral aneurysm surgeries and present one representative case in which postsurgical analysis of LSCI measurements demonstrate the potential for LSCI to detect and quantify an ischemic event. The patient is a 67-year-old female with a history of a prior aneurysm clipping who presented with a newly discovered, asymptomatic left-sided middle cerebral artery (MCA) aneurysm shown in Fig. 1. This case highlights an intraoperative ischemic event that was identified and reversed using a combination of IONM for detection of a potential ischemic event and ICGA to assess vessel patency after repositioning of the clip suspected to have caused the ischemic event.

### 2.1 Instrumentation

Custom LSCI instrumentation was attached to the neurosurgical microscope (Kinevo 900, Carl Zeiss Meditec Inc., Oberkochen, Germany) before the start of the surgery as described in previous publications.^23^^,^^24^ The instrumentation fit underneath the microscope sterile draping and did not interfere with the normal operation of the microscope. A λ=785  nm laser diode with a maximum output power of 125 mW (L785P090, Thorlabs, Newton, New Jersey, USA) was attached to a custom add-on laser adapter that mounted onto the underside of the microscope. The light reflected off a mirror and naturally diverged to a beam size of ∼4  cm at a working distance of 35 cm. The maximum irradiance was $0.01\;W/{\mathrm{cm}}^{2}$, well below the American National Standards Institute limit of $0.3\;W/{\mathrm{cm}}^{2}$ for skin at 785 nm.^35^ Back-scattered laser light was collected through the imaging optics of the microscope, then directed to an NIR-enhanced CMOS camera (acA1300–60gmNIR, Basler AG, Ahrensburg, Germany) mounted on the side of the surgeon eyepiece via a dichroic mirror; the camera operated at a frame rate of 60 fps. We used an exposure time of 3 ms based on a previous study^30^ demonstrating that exposure times of 0.5, 3, and 5 ms most closely matched the results of using a multi-exposure speckle imaging approach consisting of exposure times between 0.5 and 20 ms; we opted for 3 ms as a balance for sensitivity to both flow in visible blood vessels and microvascular perfusion measurements. A laser controller (LDC205C, Thorlabs, Newton, New Jersey, USA) ensured the laser was only on during LSCI recordings. The microscope parameters such as magnification and subsequent imaging field of view were dictated by the surgeon. For the images shown in this paper of the exposed cortex, the magnification was 3x with a range of 2 to 4x, corresponding to a field of view of 4 cm.

The IONM system used during the surgical case was the Cascade PRO IONM system controlled by the Cascade Surgical Studio software (Cadwell Industries, Kennewick, Washington, USA). The neuromonitoring modalities used with the system were somatosensory-evoked potentials (SSEPs), motor-evoked potentials (MEPs), electroencephalography (EEG), auditory brainstem responses (ABRs), and train of four (TOF). For comparison with LSCI recordings, we used transcranial electric motor-evoked potentials (TCeMEPs) from the right side, shown in Fig. S2A, because the IONM staff alerted the surgeon of deficits in this recording during the surgery.

To perform indocyanine green (ICG) video angiography, imaging was performed with the Kinevo 900 neurosurgical microscope feature “Zeiss Infrared 800” (Carl Zeiss Meditec Inc., Oberkochen, Germany). Indocyanine green was administered intravenously as a 5 mL bolus at a dosage of 2.5  mg/mL ICG (Diagnostic Green LLC, Farmington Hills, Michigan, USA) dissolved in 10 mL of sterile water.

### 2.2 Surgical Sequence

**Fig. 2:**
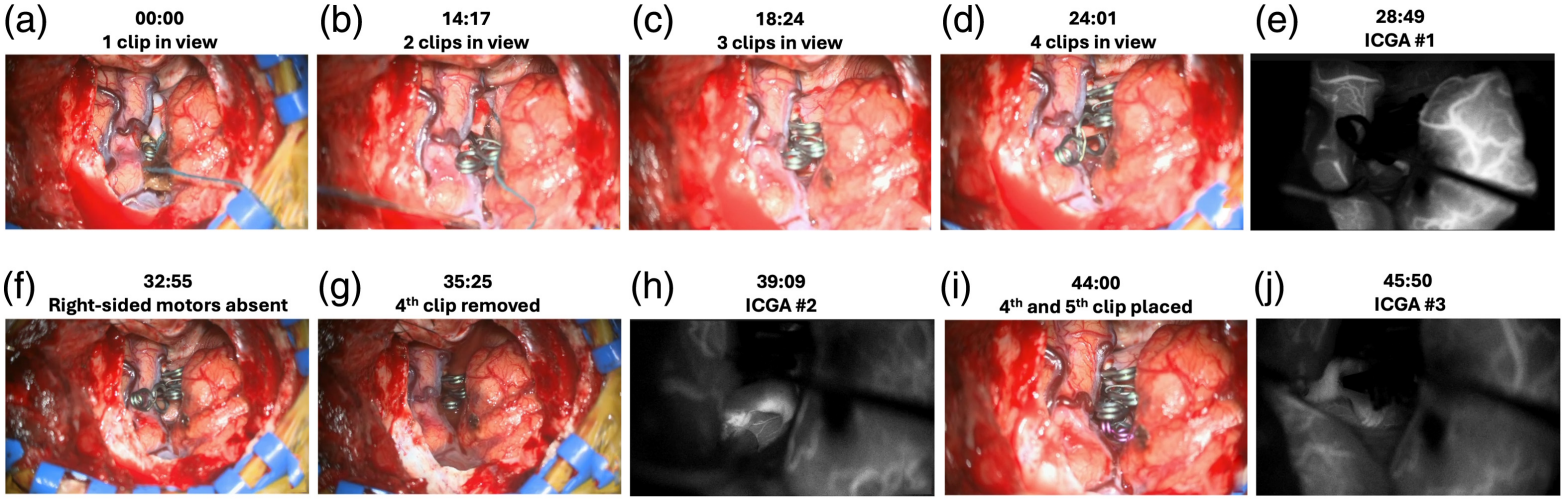
Intraoperative images detailing the surgical sequence and corresponding angiographic assessments. The sequence depicts the placement of the first three permanent clips (a-c), followed by the application of a fourth clip at 24:01 (d), which likely induced developing ischemia, followed by the first indocyanine green angiography (ICGA) assessment at 28:49 (e). The view of the surgical field at the time of the intraoperative neuromonitoring (IONM) alert (32:55) is shown in panel (f), with the subsequent removal of the clip to restore blood flow at 35:25 shown in panel (g). A second ICGA at 39:09 (h) confirmed patency of vessels surrounding the aneurysm. Two more clips were placed (i) and a third and final ICGA at 45:50 confirming successful aneurysm obliteration (j).

For describing the surgical time sequence, we use relative time where we denote time 00:00:00 (HH:MM:SS) as the first LSCI recording, occurring when the first aneurysm clip was placed. Figure 2 illustrates the surgical sequence, beginning with the placement of the first three permanent clips [Figs. 2(a)–Figs. 2(c)]. A fourth clip was then placed at a surgical time of 24:01 to achieve full obliteration of the aneurysm [Fig. 2(d)]. The first intraoperative ICGA was subsequently performed at 28:49 [Fig. 2(e)]. Minutes later at 32:55, IONM signaled a critical compromise of the right-sided motor pathways (Fig. S2A), indicating developing ischemia [Fig. 2(f)]. Suspecting the fourth clip was causing the issue, the operating surgeon promptly removed the fourth clip at 35:25 [Fig. 2(g)]. A second ICGA was performed at 39:09 [Fig. 2(h)] to evaluate whether removing the fourth clip successfully restored blood flow to three critical perforator vessels. The procedure concluded with the careful re-application of the fourth clip, the addition of a fifth clip [Fig. 2(i)], and a third and final ICGA at 45:50 to evaluate successful aneurysm obliteration and patency of vessels surrounding the aneurysm [Fig. 2(j)].

### 2.3 Post-Surgery Data Analysis

**Fig. 3:**
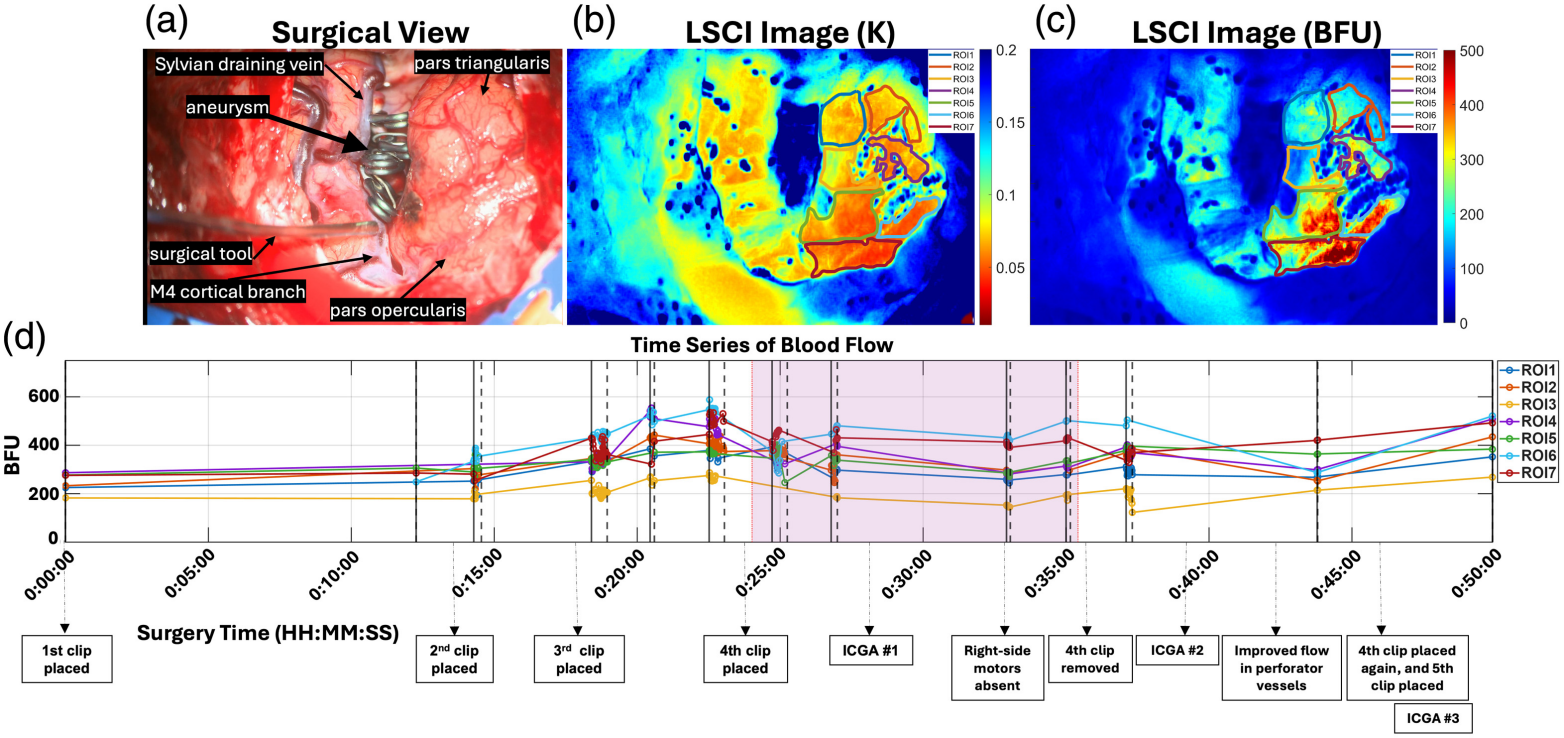
Continuous blood flow tracking in ROIs 1 to 7 throughout aneurysm clipping surgery. (a) A white light camera image showing the surgical field. (b, c) Corresponding LSCI images showing K values and BFU, respectively, with ROIs 1 to 7 outlined on the right-sided cortical surface corresponding to the inferiolateral frontal lobe. We note that the color axes are flipped between panels (b) and (c) such that red indicates high flow in both panels. (d) Time series of blood flow in the ROIs for which each point is an average of 3.5 s of LSCI measurements. The shaded magenta region indicates the ischemic period (24:01 to 35:25).

**Fig. 4:**
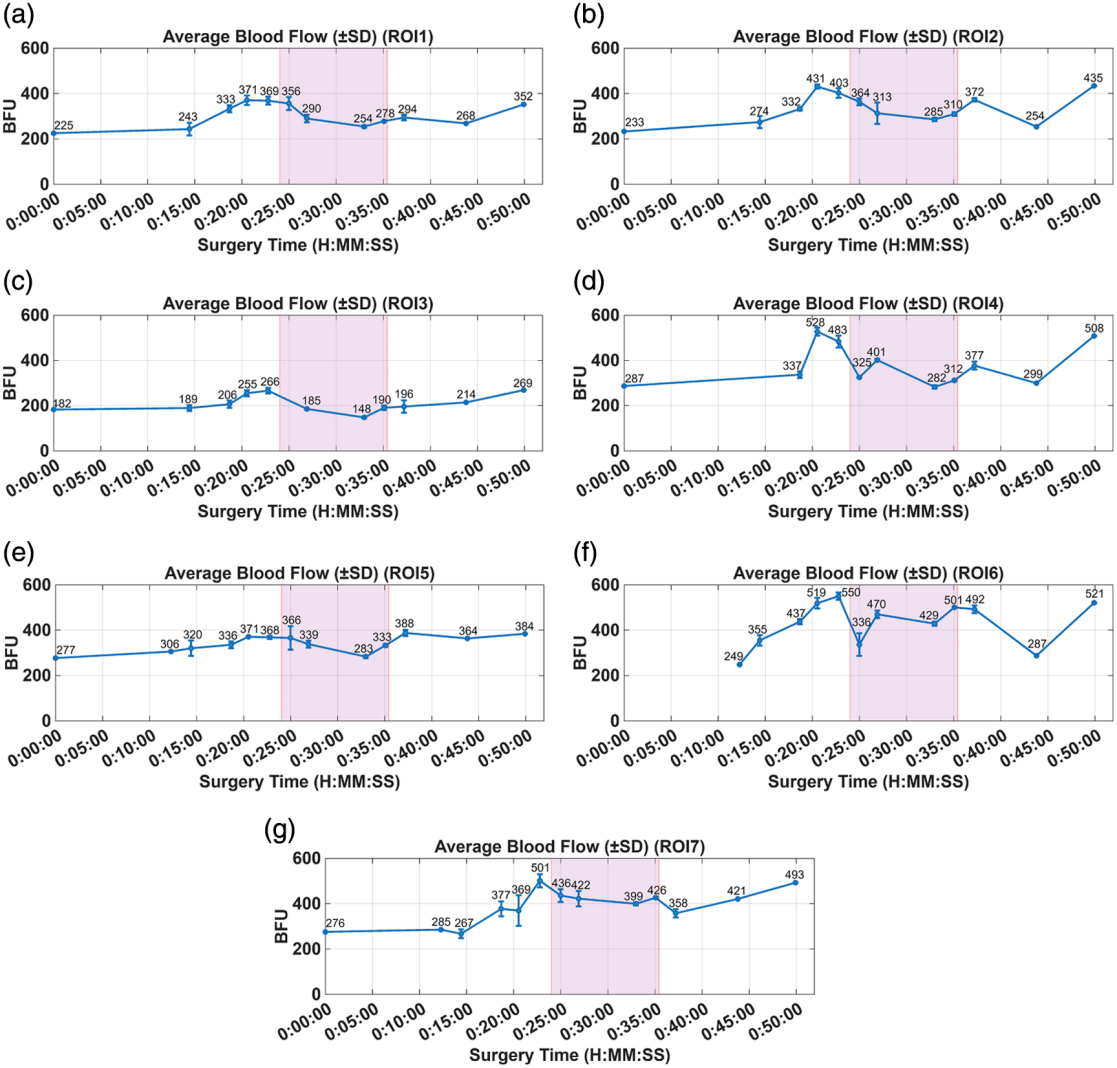
Time series of the averaged blood flow for each ROI depicted in Fig. 3(b). Plots in panels (a)–(g) correspond to ROI 1 to 7, respectively. These plots are derived by averaging the data in Fig. 3(d). The error bars represent the standard deviation.

**Fig. 5:**
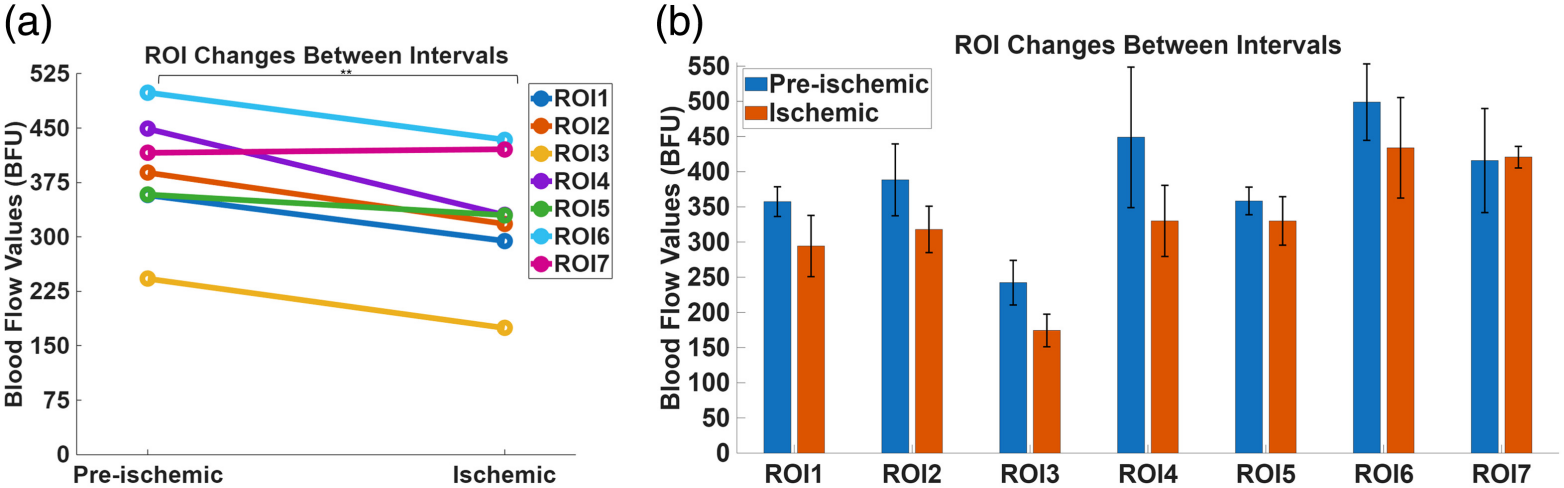
Comparison of mean cortical perfusion in regions of interest (ROIs) 1 to 7 between the pre-ischemic and ischemic period. The overall decrease in perfusion was found to be highly significant (p<0.0001).

**Fig. 6:**
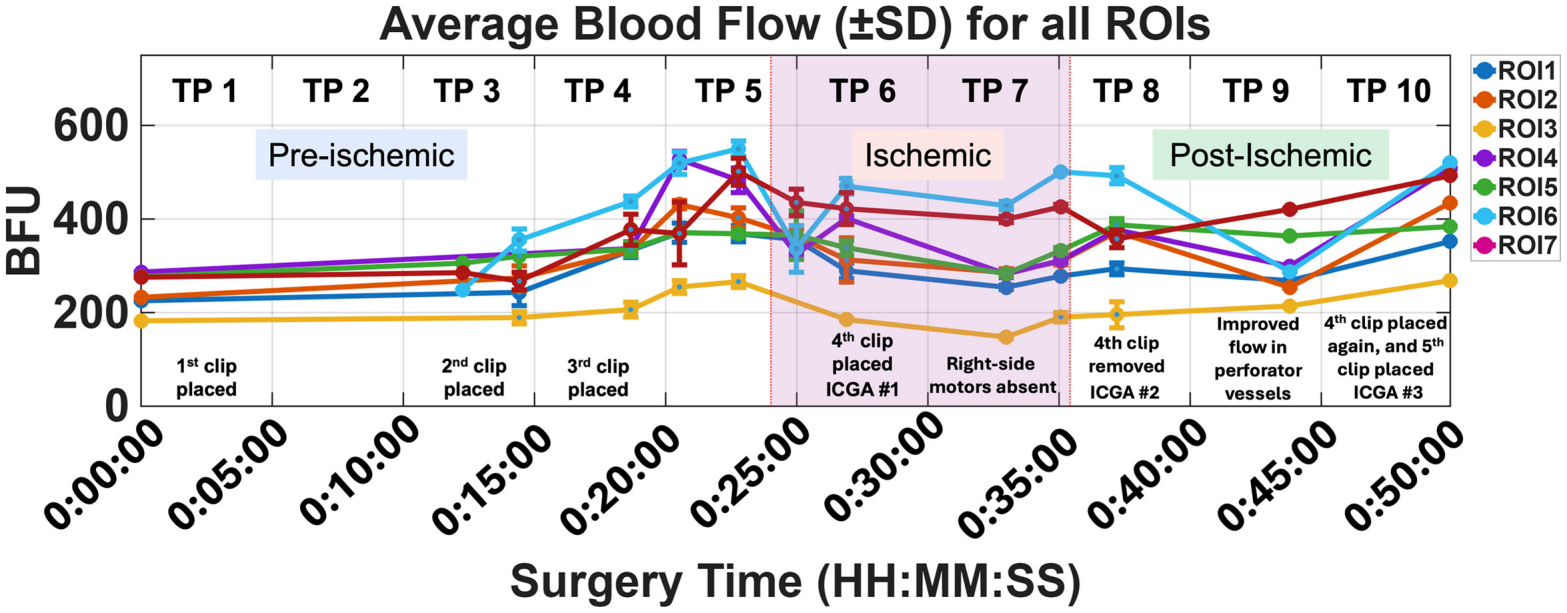
Visualization of mean cortical perfusion throughout surgery for ROIs 1 to 7. The overall decrease in perfusion between the pre-ischemic and ischemic periods was found to be highly significant (p<0.0001). TP = time period.

The microscope-integrated LSCI system assessed tissue perfusion by capturing raw speckle images and converting them to LSCI images by calculating the spatial speckle contrast, K, within a 7×7 pixel moving window according to the equation $K=\sigma_{s}/I$ where $\sigma_{s}$ is the spatial standard deviation and I is the average intensity within the region. For postsurgical analysis, we used custom MATLAB software (R2024a, The MathWorks, Inc., Natick, Massachusetts, USA) to generate perfusion plots as shown in Figs. 3, 4, 5, and 6, and Figs. S1–S3 in the Supplementary Material. To begin an analysis sequence, a single representative image was created by temporally averaging speckle contrast frames over a window duration of 0.5 to 1 s. Then, regions of interest (ROIs) were manually drawn by the user as freehand polygons on the cortical surface. Each ROI was defined to encompass a portion of a single cortical gyrus and was bounded by visible surface vessels. The ROIs are located in the inferiolateral frontal lobe and measure ∼0.5  cm in diameter (Fig. 3). ROIs 1 to 4 are positioned on the pars triangularis, and ROIs 5 to 7 on the pars opercularis; accordingly, ROIs 1 to 4 were grouped as class 1 and ROIs 5 to 7 as class 2 for statistical analysis. Next, ROI masks were applied to subsequent LSCI frames for which the microscope was not moved to ensure consistent anatomical sampling throughout the entire monitored period. This was verified by plotting the LSCI-derived blood flow signal as a function of time within each ROI in 3.5 s time windows and then visually inspecting the waveform to determine whether it exhibited consistent pulsatility corresponding to the cardiac cycle; any sudden transient drops may be indicative of obstruction of the ROI by the surgeon’s hand or a surgical tool. If inconsistent pulsatility or a sudden spike was observed, we examined the LSCI video and surgical view video during this 3.5 s time window to determine if any part of the ROI was obstructed. If any part of the ROI was obstructed at any time in the 3.5 s time window, the data for the entire 3.5 s time window for the obstructed ROI was excluded from analysis (Fig. S1 in the Supplementary Material).

For each individual LSCI frame in the time series analysis, a single mean speckle contrast value was calculated for each ROI by first calculating K for each pixel in the image using a 7×7  pixel sliding window and then averaging the K values of all pixels contained within that ROI. This single mean K value was then converted to blood flow units (BFU) using the formula $\mathrm{BFU}=1/K^{2}$, providing a relative measure of blood flow for that specific ROI at each discrete time point.^15^ Although $1/K^{2}$ is commonly referred to as blood flow index (BFI), we intentionally use the term BFU to emphasize the relative, semi-quantitative measurements in arbitrary units rather than absolute or calibrated flow; our goal is also to provide a practical and intuitive unit for intraoperative communication with surgeons while avoiding implication of absolute flow quantification. Using frame-by-frame calculations of BFU, we created a time-series of BFU for each ROI, which was then plotted against the corresponding acquisition time extracted from the system’s timing files to accurately visualize perfusion changes in specific cortical regions. We chose to display perfusion maps in BFU because the relationship $\mathrm{BFU}=1/K^{2}$ is widely used in LSCI applications; however, we acknowledge that the relationship can underestimate flow changes.^28^ Furthermore, BFU is sensitive to instrumentation parameters unrelated to blood flow, including microscope magnification which affects the speckle-to-pixel size ratio and thus K, and therefore, BFU values should only be compared across time points acquired at consistent imaging conditions.

### 2.4 Statistical Methods

A mixed effects model was employed to explore association of blood flow changes between physiological periods. We defined three physiological periods based on an iatrogenic ischemic event initiated by the placement of the 4th clip at a time of 24:01 and removed by the surgeon at 35:25. Thus, we defined the pre-ischemic period from time 00:00 to 24:00, the ischemic period as 24:01 to 35:25, and the post-ischemic period as 35:26 to 49:55. To examine if LSCI could be used in real-time during surgery, we also used the mixed effects model to explore association of blood flow changes in successive time periods (TP) of 5 min windows such that TP1 = 00:00 to 04:59, TP2 = 05:00 to 09:59, TP3 = 10:00 to 14:59, TP4 = 15:00 to 19:59, TP5 = 20:00 to 24:59, TP6 = 25:00 to 29:59, TP7 = 30:00 to 34:59, TP8 = 35:00 to 39:59, TP9 = 40:00 to 44:59, and TP10 = 45:00 to 49:59. We note no LSCI measurements of cortical perfusion were made in TP 2 (05:00 to 09:59) because the surgeon was visualizing the aneurysm at high magnification (∼7X) through the microscope during TP 2, and thus, there was no visualization of the cortex during TP 2. We also examined if blood changes were associated with anatomical groupings of ROIs for which we grouped together ROIs 1 to 4 (class 1) on the pars triangularis and ROIs 5 to 7 (class 2) on the pars opercularis. Fixed effects (time and ROI) were estimated for each of these variables with random effects by ROI. Data for blood flow were normalized to baseline blood flow at time 00:00:00 for classes 1 and 2. Differences in mean normalized blood flow for classes 1 and 2 are reported in Table 1 with two classifications of time: the first uses our definition of pre-ischemic, ischemic, and post-ischemic periods based on the iatrogenic ischemic event initiated by the placement of the 4th clip at a time of 24:01, and the second uses our definition of time periods (successive 5-min time windows) such that no knowledge about the surgical events influences this time classification. Pairwise contrasts between time classifications (pre-ischemic, ischemic, and post-ischemic; and successive 5-min time windows TP1 to TP10) were derived from the mixed effects model using estimated means. Adjusted means represent model-estimated predicted means for each class, controlling for random effects by ROI. Bonferroni correction was applied to account for multiple simultaneous pairwise comparisons. LOESS (locally estimated scatterplot smoothing) smoothed (smoothing factor 0.7) trends with predicted and actual normalized blood flow are reported in Fig. S3 in the Supplementary Material. Fixed effects estimates are displayed in Table S1 in the Supplementary Material. RStudio with R (v 4.5.0, R Core Team, R Foundation for Statistical Computing, Vienna, Austria) was used for this analysis with relevant packages [readxl; lubridate, hms, lmerTest; emmeans; dplyr; ggplot2].

To objectively identify moments of statistically significant transition in cortical perfusion independent of known surgical events, changepoint analysis was performed on normalized blood flow data pooled across all ROIs.^36^ The analysis was performed using the “changepoint” package in R. The changepoint model estimated changes in mean and variance (cpt.meanvar) using the Pruned Exact Linear Time (PELT) algorithm with the Modified Bayesian Information Criterion (MBIC) as the penalty criterion. The MBIC penalty does not define significance via a p-value threshold; rather, a changepoint is retained when adding it reduces the penalized cost function sufficiently, with the penalty controlling against spurious changepoint detection by penalizing model complexity.

## 3 Results

### 3.1 Continuous Blood Flow Tracking and Artifact Removal

During the surgery, an IONM alert occurred at 32:55 indicating the onset of an ischemic event. We aimed to evaluate whether LSCI perfusion tracking correlated with the IONM alert by evaluating the LSCI-derived perfusion data throughout the surgical case. Figure 3 illustrates blood flow tracking across the duration of the surgical procedure from time points at which the entire exposed cortical surface was within the field of view. During these acquisitions, the operative microscope magnification was ∼3X or lower; at other time points, higher magnification was used while the surgeon performed microsurgical maneuvers. The white light camera image provides anatomical context and the corresponding LSCI image depicts perfusion in both speckle contrast (K) and blood flow units (BFU), with ROIs 1 to 7 overlaid on the inferiolateral frontal lobe. The ROIs were drawn with polygon shapes to capture cortical gyri while avoiding specular reflections which appear as small blue dots in LSCI images. Time-resolved perfusion signals from these ROIs were successfully recorded throughout surgery, enabling assessment of blood flow dynamics at multiple time points. The shaded magenta region in the graphs is the ischemic period which we define as beginning at 24:01 when the fourth clip was initially placed and ending at 35:25 when the fourth clip was removed. Figure 3(d) displays the LSCI-derived BFU measurements and demonstrates a decrease in perfusion during the ischemic period. Each data point is the average of 3.5 s of LSCI measurements. To aid the reader in showing when LSCI measurements were able to be used for at least 1 ROI when the entire exposed cortical surface was within the field of view, we added vertical lines seen in Fig. 3(d): straight vertical lines indicate the starting point and dashed vertical lines denote the ending point of grouped LSCI measurements.

### 3.2 Individual ROI Blood Flow Analysis

Figure 4 shows the ROI average value of blood flow from Fig. 3(d) for which consecutive data during which the microscope was not moved [as indicated between the vertical and dashed lines in Fig. 3(d)] is averaged together. Figure 4 shows a trend of a decrease in blood flow across all ROIs during the start of the ischemic period. The error bars show the standard deviation of the BFU measurements for each group of data that was averaged. The panels in Figs. 4(a)–4(g) correspond to ROIs 1 to 7, respectively. The decrease in blood flow in each ROI at the start of the ischemic period suggests that the LSCI system can detect an ischemic event by measuring changes in perfusion. We also observe an increase in blood flow around 19:00 during the pre-ischemic period, which we attribute to an increase in blood pressure. The patient’s continuous vital signs for hemodynamic and respiratory data, shown in Fig. S4 in the Supplementary Material, indicates an increase in blood pressure at 19:00.

Figure 5 shows the changes in blood flow units for each ROI between the pre-ischemic and ischemic period for which a single representative value was calculated for each ROI by averaging from 00:00 to 24:01 for the pre-ischemic period and from 24:01 to 35:25 for the ischemic period (shaded magenta region in Figs. 3–Figs. 4). The analysis details the change in mean perfusion for each region: ROI 1 decreased from 357.4 to 294.5 BFU (17.6% reduction), ROI 2 decreased from 388.4 to 317.9 BFU (18.1% reduction), ROI 3 decreased from 242.4 to 174.4 BFU (28.0% reduction), ROI 4 decreased from 449.0 to 330.1 BFU (26.5% reduction), ROI 5 decreased from 358.4 to 330.0 BFU (7.9% reduction), and ROI 6 decreased from 498.9 to 434.0 BFU (13.0% reduction). By contrast, ROI 7 shows a slight increase from 415.9 to 420.8 BFU (a 1.2% increase). To determine the most appropriate statistical method for comparing these averaged values, the set of paired differences between these pre-ischemic and ischemic average values was first evaluated for normality using a Lilliefors test.^37^ The test confirmed that the differences were consistent with a normal distribution, thereby justifying the use of a parametric paired t-test^38^ for the primary comparison. Despite the variation in ROI 7, the paired t-test on the complete dataset (ROIs 1 to 7) yielded a p value of 0.007 which is highly significant (p<0.01). This provides strong evidence that the temporary vessel occlusion induced a statistically significant reduction in cortical blood flow across the monitored area. A more exhaustive statistical analysis using a mixed effects model was used for all LSCI perfusion measurements as described next.

### 3.3 Statistical Significance of Blood Flow Decrease during Ischemic Period

To test whether the observed decrease in blood flow during the ischemic period was statistically significant, we used a mixed effects model (described in Sec. 2.4) to explore association of blood flow changes between the pre-ischemic, ischemic, and post-ischemic periods. We found a statistically significant difference between the pre-ischemic and ischemic period (p<0.0001) in both ROIs 1 to 4 and 5 to 7. Table 1 shows the adjusted estimated mean differences between the pre-ischemic and ischemic period, and the ischemic and post-ischemic periods. Negative estimated mean differences are bolded and indicate a reduction of blood flow. In Table 1, we also examine the estimated mean differences in successive 5-min time windows for ROIs 1 to 4 and 5 to 7, for which we observed multiple significant associations between blood flow within time periods and ROI regions (p<0.05). To evaluate if, hypothetically, real-time LSCI measurements would be able to detect a significant reduction in perfusion before IONM, we examined the estimated mean differences between successive 5-min time windows (“Time Periods”). We observed a statistically significant (p<0.0001) negative estimated mean difference (indicating a reduction in blood flow) between time period 6 (25:00 to 29:59) and 7 (30:00 to 34:59); the fourth clip was placed at 24:01 and the IONM alert occurred at 32:55. Interestingly, we observe a negative estimated difference between time period 5 (20:00 to 24:59) and 6 (25:00 to 29:59) for ROIs 1 to 4; however, the p-value is 0.0788 which we considered not significant. When results are stratified by region, we observe p value differences for nearly all time periods for ROIs 1 to 4 (class 1). However, ROIs 5 to 7 (class 2) do not consistently demonstrate these same changes with period 5 to 6, 6 to 7, and 9 to 10 showing no statistical difference in perfusion. Table S1 in the Supplementary Material shows the longitudinal mixed model results predicting normalized perfusion showing statistical differences are found for periods with differences in trend by ROI.

**Table 1:** Adjusted estimated predicted mean differences for physiological periods and time periods across region of interest (ROI) for ROIs 1 to 4 (class 1) and ROIs 5 to 7 (class 2).

Contrast for evaluating statistical significance: end period – start period	ROI 1 to 4 (class 1)	p value	ROI 5 to 7 (class 2)	p value
	Estimated mean difference (95% CI)		Estimated mean difference (95% CI)	
Ischemic – pre-ischemic	−**0.222** (−0.288, −0.156)	<0.0001	−**0.299** (−0.373, −0.224)	<0.0001
Post-ischemic – ischemic	0.140 (0.060, 0.220)	0.0016	−**0.125** (−0.234, −0.015)	0.0730
Time period 3 - time period 1	0.079 (0.072, 0.230)	1.0000	0.098 (−0.104, 0.300)	1.0000
Time period 4 - time period 3	0.225 (0.155, 0.294)	<0.0001	0.209 (0.139, 0.279)	<0.0001
Time period 5 - time period 4	0.349 (0.308, 0.389)	<0.0001	0.336 (0.280, 0.393)	<0.0001
Time period 6 - time period 5	−**0.114** (−0.188, −0.041)	0.0788	0.028 (−0.062, 0.118)	1.0000
Time period 7 - time period 6	−**0.279** (−0.346, −0.212)	<0.0001	−**0.115** (−0.204, −0.025)	0.4144
Time period 8 - time period 7	0.145 (0.060, 0.230)	0.0297	0.166 (0.068, 0.264)	0.0306
Time period 9 - time period 8	−**0.198** (−0.301, −0.094)	0.0061	−**0.103** (−0.238, −0.033)	1.0000
Time period 10 - time period 9	0.544 (0.377, 0.711)	<0.0001	0.425 (0.232, 0.617)	0.0006

To aid the viewer in visualizing the perfusion changes across the defined time periods 1 to 10, Fig. 6 shows the perfusion changes for ROIs 1 to 7 throughout the surgery with both the relative surgical time and time periods 1 to 10 outlined. Each data point represents the mean of continuously acquired measurements within the corresponding time window [Fig. 3(d)].

**Fig. 7:**
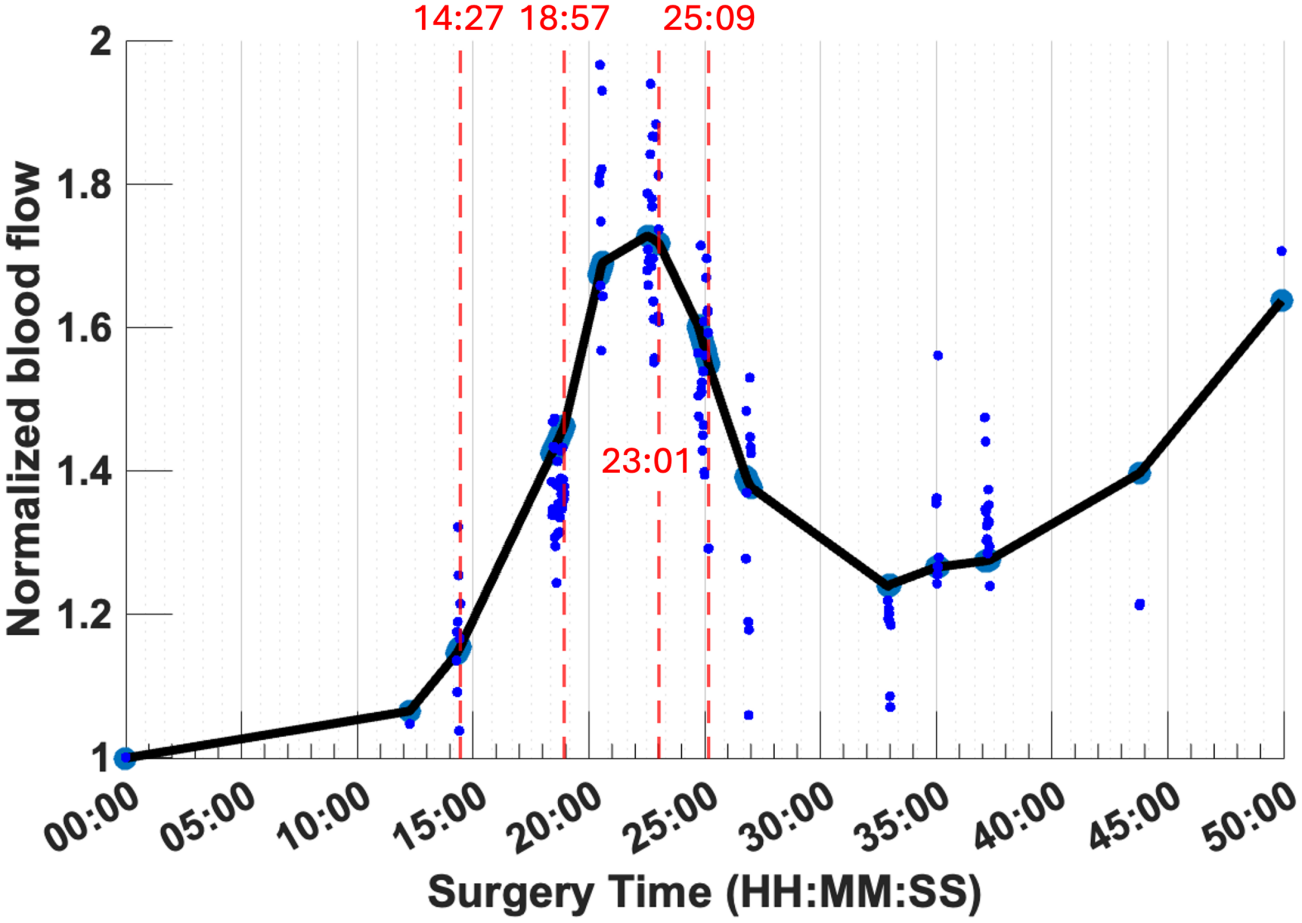
Plot of normalized blood flow aggregated across all regions of interest (ROIs) throughout the surgical case (large blue points) with LOESS-smoothed trend (black line, smoothing factor = 0.7) and individual data points (small blue points). Vertical red dashed lines indicate statistically significant changepoints at 14:27, 18:57, 23:01, and 25:09. The first two changepoints (14:27 and 18:57) reflect successive increases in blood flow during the pre-ischemic period, whereas the changepoints at 23:01 and 25:09 correspond to decreases coinciding with the ischemic period (24:01 to 35:25). The changepoint at 23:01 represents a modest departure from the local mean; the more robust reduction at 25:09 was detected 1 min and 8 s after the fourth clip placement at 24:01, suggesting that LSCI detected developing ischemia nearly 8 min before electrophysiological monitoring.

To further evaluate whether real-time LSCI measurements could detect a significant reduction in perfusion prior to IONM alerting, we performed changepoint analysis of normalized blood flow aggregated across all ROIs (Fig. 7). Four statistically significant changepoints were identified at 14:27, 18:57, 23:01, and 25:09. The first two changepoints (14:27 and 18:57) correspond to successive increases in blood flow during the pre-ischemic period. The changepoints at 23:01 and 25:09 correspond to decreases in blood flow. The changepoint at 23:01 is characterized by tightly clustered surrounding data and a small standard error, suggesting it reflects a modest but statistically detectable departure from the local mean rather than a large physiological shift; the changepoint at 25:09 is a more robust indicator of a meaningful reduction in blood flow. Notably, the fourth clip was placed at 24:01, indicating that the changepoint at 25:09 was detected 1 min and 8 s following clip placement. By contrast, the IONM alert did not occur until 32:55 — 8 min and 54 s after the placement of the fourth clip. This suggests that LSCI detected the developing ischemia nearly 8 min before IONM. These findings corroborate the longitudinal mixed effects model results presented in Table 1, which identified comparable transitions in blood flow across ROIs, collectively providing evidence that real-time LSCI perfusion measurements can detect developing ischemia substantially earlier than conventional IONM.

## 4 Discussion

This study demonstrates the potential for continuous real-time monitoring of cortical perfusion with LSCI during cerebral aneurysm clipping surgery. The primary finding is the detection and characterization of an iatrogenic ischemic event initiated by the placement of the 4th clip at 24:01. This action marked the beginning of the ischemic period (24:01 to 35:25), during which LSCI measurements show a decrease in blood flow in ROIs 1 to 6 (Figs. 4–Figs. 5, Figs. 6). In our postsurgical analysis, LSCI was able to detect this developing ischemia at 25:09, only 1 min and 8 s after the placement of the fourth clip, when performing a changepoint analysis. In our mixed effects model, the decrease in blood flow was not statistically significant until time period 7 (30:00 to 34:59) in both ROIs 1 to 4 and 5 to 7. During the surgery, IONM staff alerted the operating surgeon that the “right side motors are out” at 32:55. This clinical alert of a neurological deficit aligns precisely with the time of the most severe perfusion drop measured by LSCI, providing strong evidence of the technology’s accuracy and clinical relevance. The changepoint analysis of LSCI-derived normalized blood flow changes suggests this approach can detect a downward trajectory of blood flow that may potentially lead to ischemia minutes before IONM. Further work and validation are needed to implement a robust statistical method that is highly sensitive to developing ischemia during neurosurgical procedures to inform a clinically actionable event by the surgeon.

The semi-quantitative output of LSCI provides more objective perfusion metrics beyond qualitative visual inspection of the cortical surface by the surgeon under white-light microscopy.^19^^,^^39^ The drop in perfusion during the ischemic period was visually apparent in LSCI images and confirmed to be highly significant by a paired t-test (p=0.007) and mixed effects model (p<0.0001) with an estimated drop of 22.2% in ROIs 1 to 4 (95% CI, 15.6% to 28.8%) and 29.9% in ROIs 5 to 7 (95% CI, 22.4% to 37.3%). As detailed in the Results, the magnitude of this event varied regionally and over time, with perfusion decreases in six of the seven ROIs, including a maximal drop of 28.0% in ROI 3. The subsequent removal of the offending 4^th^ clip at 35:25 initiated the reperfusion phase. The ability to reliably extract these physiological signals was possible due to our microscope-integrated LSCI system which visualized cortical blood flow during periods when the exposed cortical surface was within the microscope field of view, generally at microscope magnifications of ∼3X or lower. Further, we also developed a methodology for removing imaging artifacts, which will be vital to automate in real-time for using LSCI effectively in the dynamic environment of an operating room.

The LSCI measurements immediately prior to the ischemic period show an increase in blood flow which we attribute to an increase in blood pressure at 19:00 (Fig. S4 in the Supplementary Material). This finding demonstrates that LSCI may also be useful for monitoring how induced changes of blood pressure affect blood flow and tissue perfusion on the cortical surface. Notably, this blood pressure-driven elevation in perfusion also explains why LSCI-measured blood flow at the time of the IONM alert (32:55) remained ∼25% above the baseline at a surgery time of 00:00.

The study includes multiple limitations that we aim to overcome in future studies. One, the analysis was done after surgery; future work will aim to perform this analysis in real-time during surgery. Two, we used a single camera exposure for performing LSCI measurements, which may underestimate flow changes.^25^ However, even with the potential for underestimating the flow changes during the ischemic period, we still observed a statistically significant reduction in blood flow using a paired t-test analysis (Fig. 5), mixed effects model (Table 1, and Table S1 and Fig. S3 in the Supplementary Material), and changepoint analysis (Fig. 7). Because this analysis was based on a single patient, it is reasonable to be conservative when generalizing these findings. However, these results demonstrate significant differences in blood flow over time, specifically during the ischemic episode, with differences in these trends found by region (Table 1, and Table S1 and Fig. S3 in the Supplementary Material). A third limitation is the occurrence of data gaps, such as the entirety of time period 2 (05:00 to 09:59), often caused by the surgeon performing microsurgery maneuvers at a high microscope magnification (>7X) such that only the aneurysm is in view and not the cortical surface. This limitation may be unavoidable when integrating an LSCI system on a surgical microscope because the LSCI camera field of view is dictated by the microscope imaging optics. A potential solution to keep the entire exposed cortical surface in view by the LSCI camera is to use separate imaging optics for the LSCI camera. In addition, because BFU is sensitive to instrumentation parameters such as microscope magnification, which affects the measured speckle contrast because of the change in speckle-to-pixel size ratio, frames acquired at varying magnifications cannot be directly compared; this further motivates separate imaging optics for the LSCI camera for which the magnification is held constant. In this study, we only used frames for which the entire exposed surface was in the field of view which was at 3x with a range of 2 to 4x. Furthermore, the changepoint analysis was applied to the aggregated BFU time series as a single contiguous vector ordered sequentially by acquisition time. The PELT algorithm, as implemented in the changepoint package in R, operates on an indexed sequence of observations without explicit knowledge of the time axis; gap boundaries could in principle be identified as spurious changepoints since the algorithm cannot distinguish a physiological transition from a discontinuity introduced by a data gap. The clinically relevant changepoints at 23:01 and 25:09 occur within a continuously sampled region of the data where no gaps are present, which mitigates this concern for the primary findings. Future implementations should limit data gaps or employ changepoint methods that account for irregular sampling. A fourth limitation is we only performed the mixed effects model for 300 s time period windows; future studies will aim to incorporate shorter time periods to further investigate the sensitivity of LSCI-derived perfusion measurements to developing ischemia.

The implications of these findings are significant for intraoperative monitoring of brain health during neurosurgery. Although intermittent techniques such as ICGA are used to confirm vessel patency at specific moments as demonstrated by Figs. 2(e), 2(h), and 2(j), its interpretation for tissue perfusion is qualitative and subjective, and current quantitative perfusion methods for ICGA are not yet standardized nor widely clinically adopted. By contrast, LSCI offers continuous, semi-quantitative data that can immediately detect compromised blood flow. Thus, LSCI can enable a surgeon to correct an issue with clip placement before a neurological deficit is registered by IONM or becomes permanent. The heterogeneity observed between ROIs suggests that LSCI can also provide insight into regional variations in collateral circulation. Although this single, detailed case study provides a strong proof-of-concept, future work should include a larger patient cohort to establish specific perfusion thresholds that may correspond to reversible versus irreversible ischemic damage. Such studies would be a critical step toward fully integrating LSCI as a standard of care for intraoperative perfusion monitoring.

## 5 Conclusion

This study validates LSCI as an effective, non-invasive, and semi-quantitative tool for intraoperative monitoring of tissue perfusion in real-time. We successfully demonstrated the ability of LSCI to detect and quantify an iatrogenic ischemic event corroborated by both the surgical events and an independent IONM alert. LSCI’s capacity to provide continuous feedback on tissue perfusion represents a complementary tool to IONM and ICGA. The use of LSCI during neurosurgical procedures holds considerable promise for enhancing patient safety by allowing for the early detection of compromised blood flow and confirmation of restored blood flow, thereby potentially reducing the risk of postoperative neurological deficits.

## Supplementary Material

10.1117/1.NPh13.3.035003.s01

## Data Availability

Data files analyzed during the current study are available on GitHub: github.com/MillerLabOU
