# Supplementary material for: Intraoperative tracking of tissue perfusion during cerebral aneurysm surgery with laser speckle contrast imaging: insights beyond standard intraoperative neuromonitoring for detecting ischemia
Source: Neurophotonics. 2026 Jul 7;13(3):035003. doi: 10.1117/1.NPh.13.3.035003 (PMC13340444; doi:10.1117/1.NPh.13.3.035003)
Supplement: Supplementary file 1 [file NPh_013_035003_SD001.pdf]

Supplemental Materials

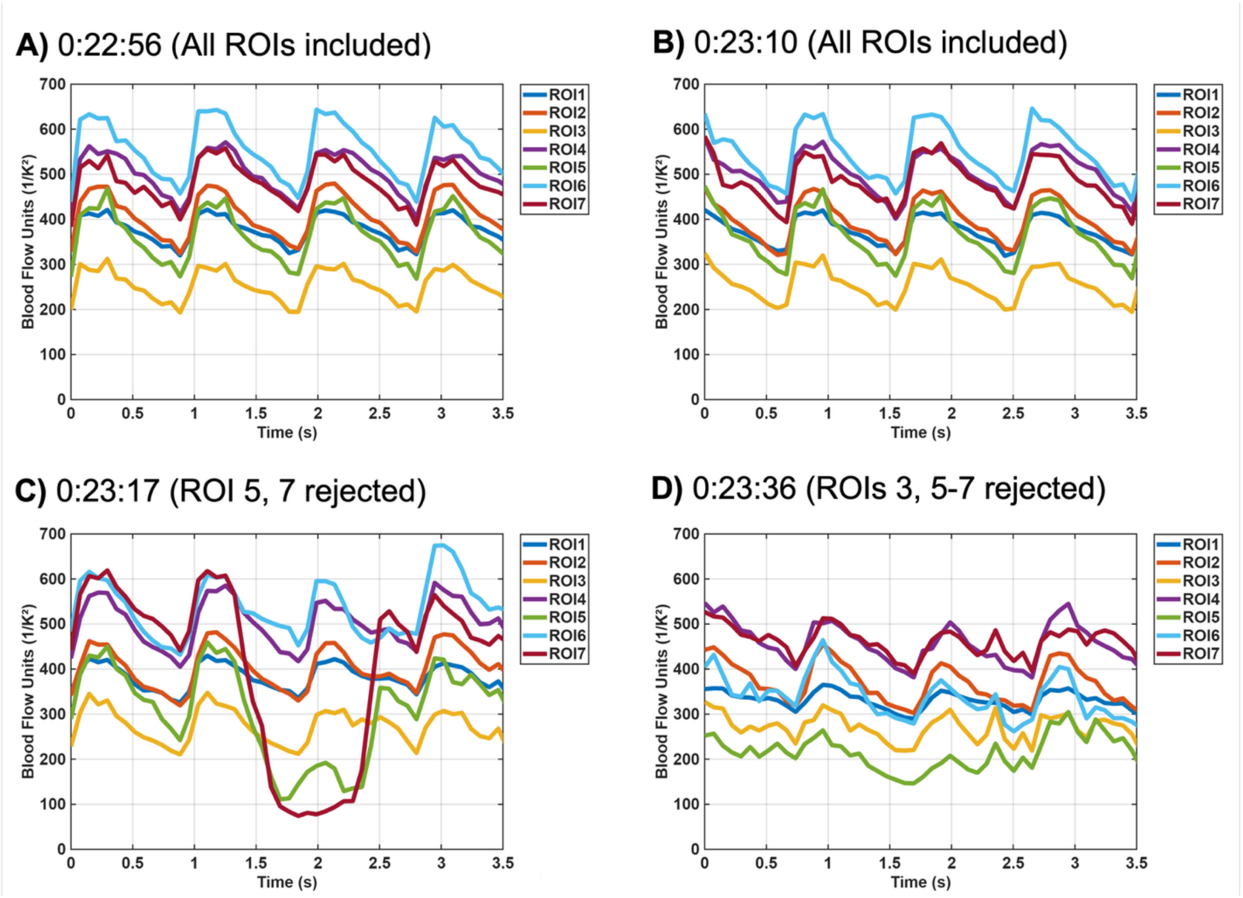

Fig. S1 Example analysis of including or rejecting time series of blood flow for ROIs 1-7 using 3.5 second time window periods. If pulsatility was not observed at a specific time for an ROI, we watched the LSCI video and surgical view video at this time point to examine if there was an obstruction of any part of the ROI, such as by the surgeon's hands or a surgical tool. **(A)** Occurring at a surgical time of 22:56, all ROIs displayed consistent pulsatile flow and were included in the analysis. **(B)** Occurring at a surgical time of 23:10, all ROIs displayed consistent pulsatile flow and were included in the analysis. **(C)** Occurring at a surgical time of 23:17, all ROIs except ROI 5 and 7 displayed consistent pulsatile flow. Upon verifying the LSCI and surgical view video, ROIs 5 and 7 contained obstructions and were rejected for the analysis. **(D)** Occurring at a surgical time of 23:36, only ROIs 1-2 and 4 displayed consistent pulsatile flow. Upon verifying the LSCI and surgical view video, ROIs 3 and 5-7 contained obstructions and were rejected for the analysis.

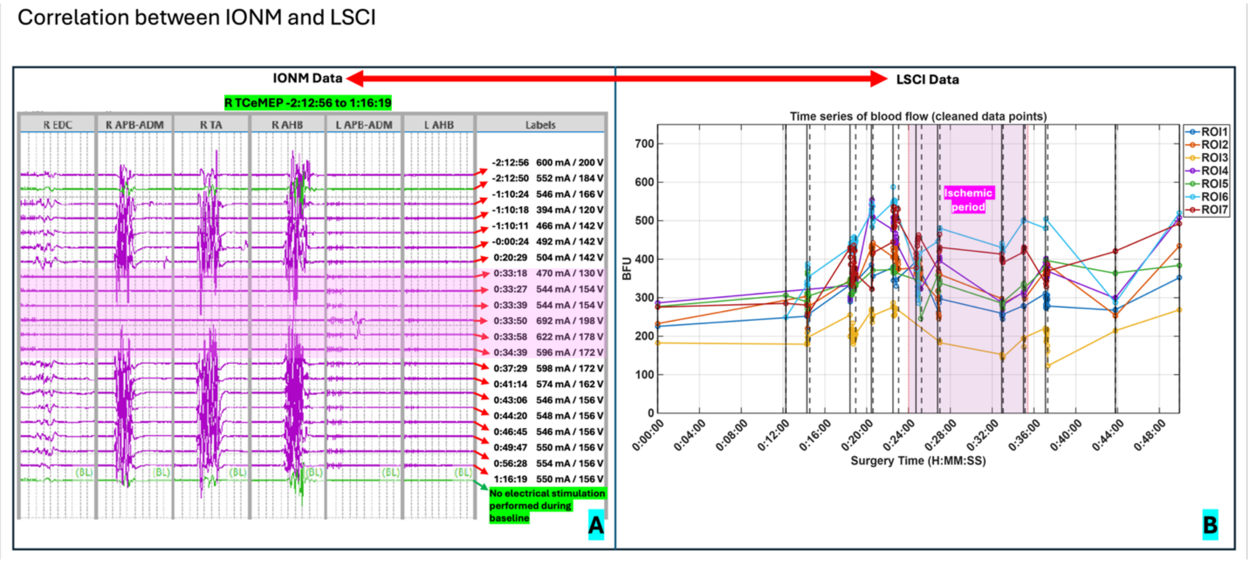

Fig. S2 (A) A summary of the patient's transcranial electric motor evoked potentials (TCeMEPs), one of the intraoperative neuromonitoring (IONM) modalities used during the surgical case. The vertical "Labels" column on the right shows the time axis with the electrical stimulation parameters used to evoke the recorded responses. The recorded responses are shown in the purple traces. The purple shaded region between the recordings 33:18 and 34:39 occurred during the "ischemic period" which we defined as between 24:01 (placement of 4th clip) and 35:25 (removal of 4th clip). The IONM staff alerted the surgeon of deficits in this recording during the surgery at 32:55 based on the lack of signal, suggesting a developing ischemia. **(B)** The corresponding laser speckle contrast imaging (LSCI) measurements shown in blood flow units (BFU) where the x-axis is in time and the shaded magenta region indicates the ischemic period. The LSCI measurements show a reduction in blood flow, demonstrating that LSCI can serve as a complementary tool to IONM.

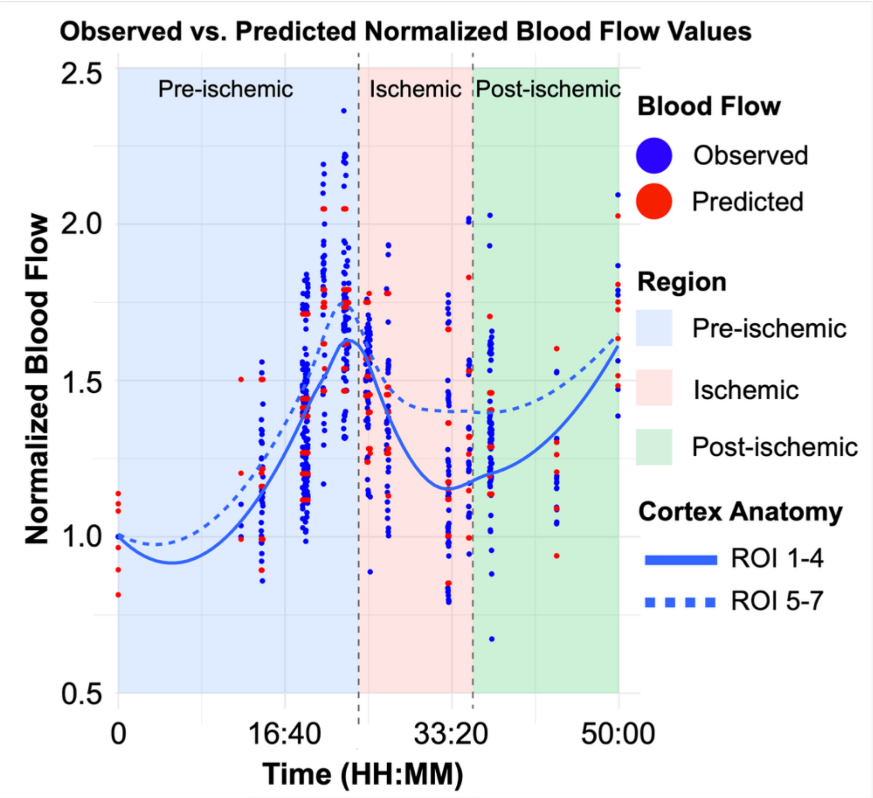

Fig. S3 Normalized blood flow values throughout the surgery for regions of interest (ROIs) 1–4 (Class 1, pars triangularis) and ROIs 5–7 (Class 2, pars opercularis). Observed values are shown with LOESS (locally estimated scatterplot smoothing) trends (smoothing factor = 0.7). Predicted blood flow values were derived from a mixed effects model with fixed effects for time period (10 successive 5-minute windows, reference = Period 1), ischemic state (pre-ischemic, ischemic, post-ischemic; reference = pre-ischemic), ROI class (Class 1 vs. Class 2), and their interactions. Full model coefficients are reported in Table S1.

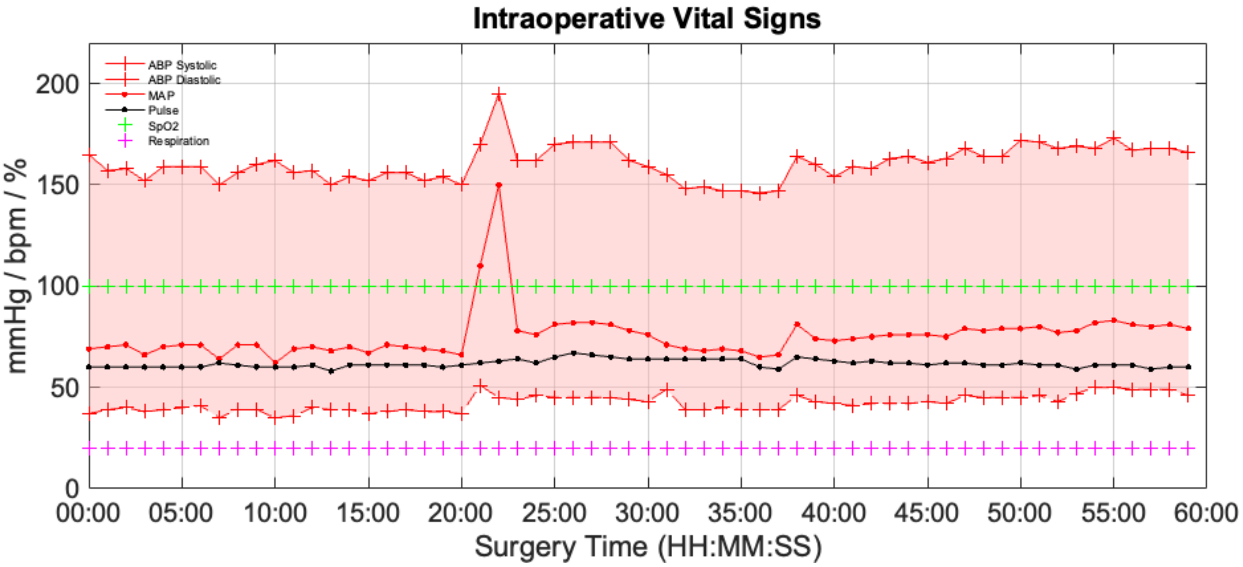

Fig. S4 Patient's continuous vital signs for hemodynamic and respiratory data during the surgical case displaying arterial blood pressure (ABP), mean arterial pressure (MAP), pulse, respiration rate, and peripheral oxygen saturation (SpO2). The x-axis units correspond to the measure: mmHg for ABP and MAP, beats per minute (bpm) for pulse and respiration, % for SpO2.

Table S1: Mixed Model Results – Prediction of Normalized Perfusion

Variable	Class	Estimate	SE	t-stat	p-value
(Intercept)		1.00	0.120	8.31	0.0000
Time period	3	0.08	0.076	1.03	0.3023
(ref = period 1)	4	0.30	0.070	4.33	< 0.0001
	5	0.65	0.071	9.23	< 0.0001
	6	0.54	0.080	6.75	< 0.0001
	7	0.26	0.080	3.23	0.0013
	8	0.40	0.084	4.79	< 0.0001
	9	0.21	0.099	2.08	0.0378
	10	0.75	0.111	6.79	< 0.0001
Ischemic	Ischemic	-0.22	0.033	-6.69	< 0.0001
(ref=Pre-Ischemic)	Post-Ischemic	-0.08	0.052	-1.57	0.1173
ROI Group	Class 1 vs 2	0.14	0.192	0.70	0.4946
Ischemic×ROI Group	Ischemic, Class 1	-0.08	0.050	-1.53	0.1263
	Post-Ischemic, Class 1	-0.34	0.085	-4.03	*0.0001*
Time period×ROI Group	Period 3, Class 1	0.02	0.128	0.15	0.8818
	Period 4, Class 1	0.00	0.121	0.03	0.9748
	Period 5, Class 1	-0.01	0.123	-0.07	0.9425
	Period 6, Class 1	0.13	0.136	0.98	0.3285
	Period 7, Class 1	0.30	0.135	2.20	*0.0282*
	Period 8, Class 1	0.32	0.140	2.27	*0.0235*
	Period 9, Class 1	0.41	0.165	2.51	*0.0123*
	Period 10, Class 1	0.29	0.181	1.63	0.1046

The “Variable” column identifies the fixed effect term in the model: “Time Period “(10 successive 5-minute windows, reference = Period 1), “Ischemic” state (pre-ischemic, ischemic, post-ischemic; reference = pre-ischemic), “ROI Group” (Class 1: ROIs 1–4 vs. Class 2: ROIs 5–7), and interaction terms (“Ischemic × ROI Group”; “Time Period × ROI Group”). The “Class” column specifies the particular level of that factor being estimated (i.e., Period 3, Ischemic, Post-Ischemic, Class 1). “Estimate” is the model coefficient (change in normalized blood flow relative to reference); SE = standard error of the estimate; t-stat = t-statistic; p-value = two-tailed p-value. Random effects: SD(ROI) = 0.1978. We note that no LSCI measurements of cortical perfusion were made in TP 2.
